# Monoamine Oxidases (MAOs) as Privileged Molecular Targets in Neuroscience: Research Literature Analysis

**DOI:** 10.3389/fnmol.2019.00143

**Published:** 2019-05-29

**Authors:** Andy Wai Kan Yeung, Maya G. Georgieva, Atanas G. Atanasov, Nikolay T. Tzvetkov

**Affiliations:** ^1^Oral and Maxillofacial Radiology, Applied Oral Sciences, Faculty of Dentistry, The University of Hong Kong, Hong Kong, China; ^2^Department of Biochemical Pharmacology and Drug Design, Institute of Molecular Biology Roumen Tsanev, Bulgarian Academy of Sciences, Sofia, Bulgaria; ^3^The Institute of Genetics and Animal Breeding, Polish Academy of Sciences, Magdalenka, Poland; ^4^Department of Pharmacognosy, University of Vienna, Vienna, Austria

**Keywords:** molecular neuroscience, monoamine oxidase, tyramine, bibliometrics, history, Alzheimer’s disease, Parkinson’s disease, depression

## Abstract

**Background**: Monoamine oxidases (MAOs) were discovered nearly a century ago. This article aims to analyze the research literature landscape associated with MAOs as privileged class of neuronal enzymes (neuroenzymes) with key functions in the processes of neurodegeneration, serving as important biological targets in neuroscience. With the accumulating publications on this topic, we aimed to evaluate the publication and citation performance of the contributors, reveal the popular research themes, and identify its historical roots.

**Methods**: The electronic database of Web of Science (WoS) Core Collection was searched to identify publications related to MAOs, which were analyzed according to their publication year, authorship, institutions, countries/regions, journal title, WoS category, total citation count, and publication type. VOSviewer was utilized to visualize the citation patterns of the words appearing in the titles and abstracts, and author keywords. CRExplorer was utilized to identify seminal references cited by the MAO publications.

**Results**: The literature analysis was based on 19,854 publications. Most of them were original articles (*n* = 15,148, 76.3%) and reviews (*n* = 2,039, 10.3%). The top five WoS categories of the analyzed MAO publications were Pharmacology/Pharmacy (*n* = 4,664, 23.5%), Neurosciences (*n* = 4,416, 22.2%), Psychiatry (*n* = 2,906, 14.6%), Biochemistry/Molecular Biology (*n* = 2,691, 13.6%), and Clinical Neurology (*n* = 1,754, 8.8%). The top 10 institutions are scattered in the United States, UK, France, Sweden, Canada, Israel, and Russia, while the top 10 countries/regions with the most intensive research on the field of MAOs are the United States, followed by European and Asian countries. More highly cited publications generally involved neurotransmitters, such as dopamine (DA), serotonin, and norepinephrine (NE), as well as the MAO-A inhibitors moclobemide and clorgyline, and the irreversible MAO-B inhibitors selegiline and rasagiline.

**Conclusion**: Through decades of research, the literature has accumulated many publications investigating the therapeutic effects of MAO inhibitors (MAOIs) on various neurological conditions, such as Alzheimer’s disease (AD), Parkinson’s disease (PD), and depression. We envision that MAO literature will continue to grow steadily, with more new therapeutic candidates being tested for better management of neurological conditions, in particular, with the development of multi-target acting drugs against neurodegenerative diseases.

## Introduction

**Figure 1:**
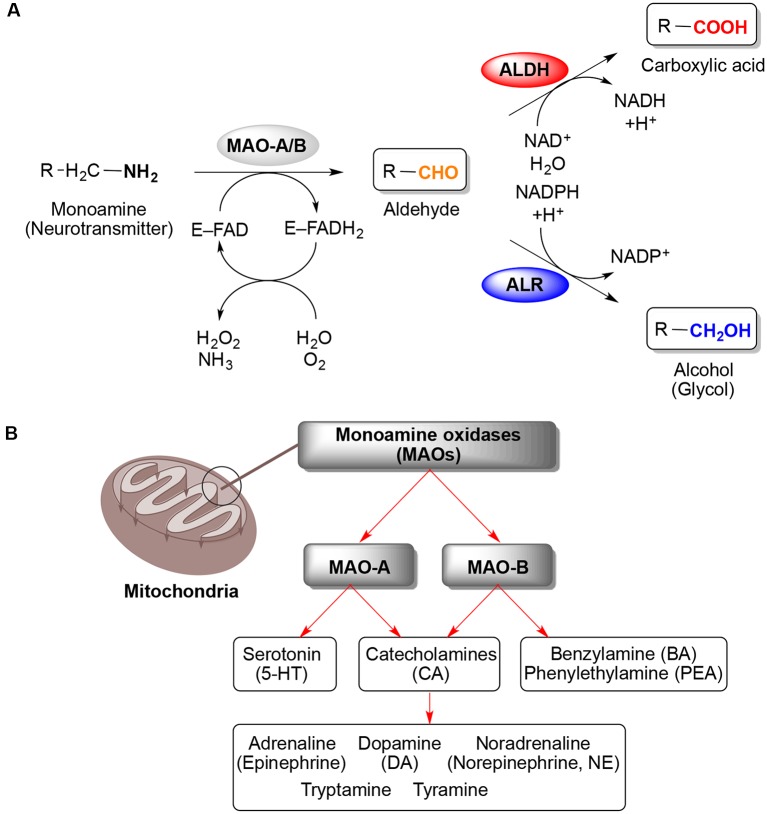
Oxidative deamination of monoamines catalyzed by monoamine oxidases (MAOs) A and B. **(A)** General reaction scheme showing the binding of a monoamine (neurotransmitter) to the flavoenzyme (E–FAD) to yield the respective aldehyde and ammonia *via* reduction of the flavin adenine dinucleotide (FAD) co-factor toward FADH_2_ (step 1), followed by conversion of the aldehyde (step 2) either to carboxylic acid *via* aldehyde dehydrogenase (ALDH) or into alcohol (glycol) by aldehyde reductase (ALR). **(B)** Localization of MAOs on the mitochondrial outer membrane and their specificities in the oxidative deamination of monoamine neurotransmitters (Maggiorani et al., 2017).

Monoamine oxidases (MAOs, EC 1.4.3.4) were discovered by Mary L.C. Hare (later known as Mary Bernheim) nearly a century ago, back in 1928 (Hare, 1928). The discovery of the first MAO, originally called tyramine oxidase, has paved the way for researchers to study the potential of MAOs as biological targets and development of therapeutics, mainly related to neurological diseases (Zeller and Barsky, 1952; Slotkin, 1999; Youdim and Bakhle, 2006; Jo et al., 2012). MAOs are flavin adenine dinucleotide (FAD) co-factor-dependent enzymes localized on the mitochondrial outer membrane that catalyze the oxidation of endogenous and xenobiotic monoamines (Figure 1A). Therefore, MAOs play an important role in the central and peripheral nervous system (CNS and PNS) by modulating the levels of monoamine neurotransmitters (Setini et al., 2005). Two isoforms are present in most mammalian tissues, MAO-A and MAO-B. Although there is ~73% identity of the protein sequences, both MAOs are important for the inactivation of various neurotransmitters but display regional differences in enzyme activity, substrate specificity, and distribution in the human brain and periphery (Shih et al., 1999; Binda et al., 2002; Castagnoli et al., 2003). For example, serotonin (5-hydroxytryptamine, 5-HT) is preferably degraded by MAO-A (Tong et al., 2013), whereas MAO-B exhibits higher affinity toward benzylamine (BA) and phenylethylamine (PEA; Youdim and Bakhle, 2006; Jo et al., 2012; Tong et al., 2013). Catecholamines such as dopamine (DA), adrenaline (epinephrine), noradrenaline (norepinephrine, NE), tryptamine, and tyramine are substrates for both MAO isoforms (Figure 1B). However, DA is mainly metabolized by MAO-B in *substantia nigra*, where MAO-B is the main distributed isoform in glial cells and the increased MAO-B activity is associated with loss of DA in the human brain (Tzvetkov et al., 2017).

**Figure 2:**
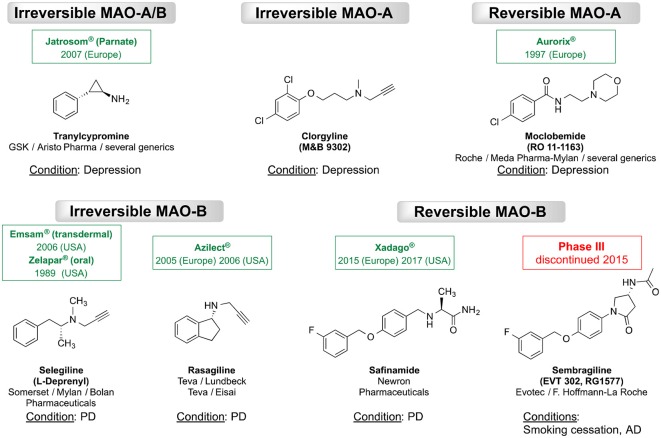
Chemical structures of the most known irreversible and reversible monoamine oxidase inhibitors MAO inhibitors (MAOIs). The international nonproprietary, brand or generic names with the approval year (for approved drug), producer/developer companies, as well as the medical conditions are indicated.

As MAOs play a key role in regulating neurotransmitter levels, altered MAO levels may associate with several neurological diseases. The abnormal MAO-A genotype is associated with Brunner syndrome (Brunner et al., 1993) and autism (Cohen et al., 2011). Furthermore, the elevated MAO-A levels may link to major depression (Meyer et al., 2006; Tong et al., 2013). Similarly, there seems to be an association between the increased MAO-B levels (~4-fold with aging) and neurodegenerative diseases, such as Alzheimer’s disease (AD) and Parkinson’s disease (PD; Saura et al., 1994; Mallajosyula et al., 2009). The preferences in substrate (neurotransmitter) affinity are essential for the different clinical significance of both MAOs, e.g., *via* inhibiting the activity either of MAO-A or MAO-B by monoamine oxidase MAO inhibitors (MAOIs). Therefore, selective inhibition of MAO-A in the human brain is an established approach for the treatment of mental disorders, while selective MAO-B inhibitors are those used for treating of PD (Riederer et al., 2004a; Yamada and Yasuhara, 2004; Tzvetkov et al., 2017). Subsequently, a number of MAOIs have been developed and approved worldwide for the treatment of neurological or psychiatric diseases. For example, the irreversible non-selective MAOIs tranylcypromine, the selective MAO-A inhibitors such as the irreversible inhibitor clorgyline and the reversible inhibitor moclobemide are used to treat depression and anxiety (for structures, see Figure 2; Riederer et al., 2004b; Tzvetkov et al., 2017). A meta-analysis reported that selective MAO-A inhibitors have a better efficacy than tricyclic antidepressants for managing atypical depression (Henkel et al., 2006). Furthermore, the irreversible MAO-B inhibitors selegiline and rasagiline (first generation MAO-B inhibitors) are approved as monotherapy for early PD or in combination with levodopa in late-stage PD (Lakhan, 2007; Fowler et al., 2015). However, it is known that irreversible MAO inhibition may cause adverse pharmacological effects and safety complications (Kumar et al., 2016). In 2015, the reversible MAO-B inhibitor safinamide has been approved as an add-on drug to levodopa or to DA agonists for the treatment of motor complications in patients with mid-to late-stage or early PD, respectively (Deeks, 2015). In contrast, the reversible MAO-B inhibitor sembragiline, a compound that was patented and investigated as a smoking-cessation agent, was discontinued in clinical phase III as a medication for the treatment of moderate AD (Borroni et al., 2017; Tzvetkov et al., 2019).

**Figure 3:**
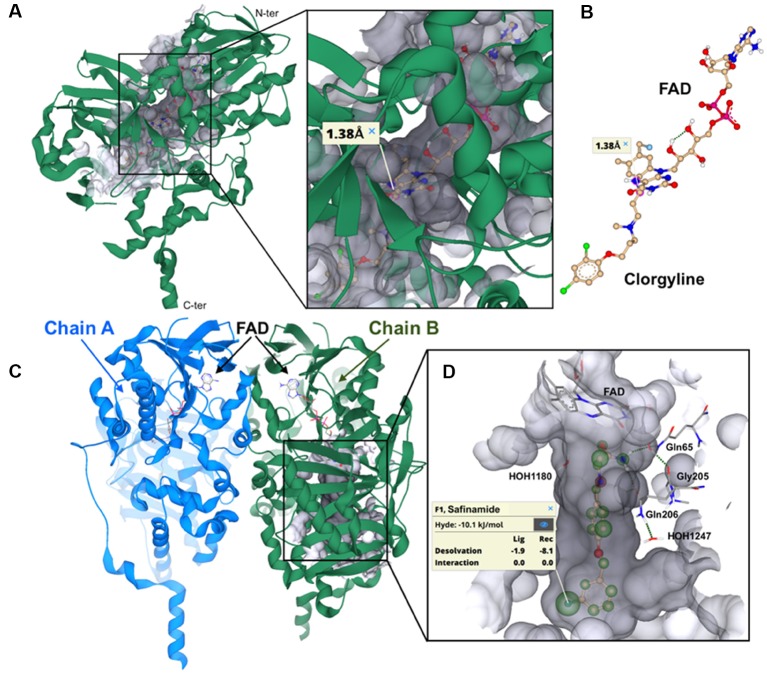
Visualization of the crystal structures of *h*MAO-A and *h*MAO-B. The binding mode of irreversible and reversible MAO inhibition on the example of the most prominent MAO-A inhibitor clorgyline (irreversible) and MAO-B inhibitor safinamide (reversible) is represented. **(A)** Ribbon representation of the co-crystallized structure of clorgyline with the monomer *h*MAO-A (PDB: 2BXS, resolution: 3.15 Å). The C-terminal membrane region and the N-terminus are depicted. The surface for binding site is colored in gray transparent. **(B)** Representation of the covalent bonding (1.38 Å) between the irreversible MAO-A inhibitor clorgyline and the FAD co-factor. **(C)** Ribbon representation of the co-crystallized structure of safinamide with *h*MAO-B (PDB: 2V5Z, resolution: 1.6 Å). The respective chain A and B of *h*MAO-B dimer with FAD are depicted. **(D)** HYDE (HYdrogen bonding and DEsolvation) analysis of desolvation effects and interactions for safinamide (off-white). HYDE visual affinity assessment as embedded in SeeSAR: green = favorable, red = unfavorable and non-colored = no relevant for affinity. The interacting amino acid residues, important water molecules and FAD are shown. Visualization and HYDE analysis were performed using the SeeSAR tool from BioSolveIT (v.8.2, 2019).

The resolution of the X-ray co-crystal structures of both human MAO-A and MAO-B with a number of irreversible and reversible inhibitors has not only gained new insight into the structure of these enzyme-ligand complexes, but also has newly inspired the research in the field of MAO inhibition as potential therapeutic approach in neurological diseases (Binda et al., 2002, 2004, 2007; De Colibus et al., 2005). Although electron paramagnetic resonance (EPR) experiments showed that human MAO-A and MAO-B isoenzymes are dimeric in their physiological forms (Kumar et al., 2016), the crystallographic studies revealed that the human MAO-A isoenzyme crystallizes as monomer (De Colibus et al., 2005; Son et al., 2008), whereas human MAO-B crystallizes as dimer (Binda et al., 2007). Furthermore, the active site of the human MAO-A consists of a single hydrophobic cavity with a volume of ~550 Å^3^, while the bipartite cavity of human MAO-B has a volume of ~700 Å^3^, divided into substrate binding site with the FAD co-factor (~400 Å^3^) and entrance hydrophobic cavity (~300 Å^3^; De Colibus et al., 2005). The X-ray structures of human MAO-A and human MAO-B complexes with the covalent (irreversible) bonded MAO-A inhibitor clorgyline and the non-covalent (reversible) MAO-B inhibitor safinamide, respectively, are showed in Figure 3.

In the current study, we aimed to analyze the research literature landscape concerning MAOs as privileged biological targets, in particular, in neuroscience from two bibliometric perspectives. First, we evaluated the publication and citation data of the literature, to identify the major contributors in terms of authors, institutions, countries/regions, and journals. By analyzing the words from titles, abstracts and keywords, we identified the hotspots of the field and revealed which themes were more investigated and cited in the period of time between 1928 and March 2019. Second, after performing the traditional citation analysis, we evaluated the cited references of the literature associated with the MAOs research. Cited reference analysis enables researchers to identify seminal publications that are important to a pre-defined body of literature, which may not be identified by traditional citation analysis due to several reasons. For example, that may be not all-time highly cited publications (only highly cited by a pre-defined body of literature, such as literature related to MAOs), but also not mentioning the exact words used by the literature search or not directly dealing with the topic of the literature search. Using this technique, we aimed to identify the historical roots and seminal references that may not be all-time highly cited but are still very important to the research field of MAOs.

## Materials and Methods

### Data Source

In March 2019, we assessed the Web of Science (WoS) Core Collection electronic database, a multidisciplinary online database hosted by Clarivate Analytics, to search with the following string: TOPIC = (“monoamine oxidase*” OR “MAO-A*” OR “MAO-B*” OR MAOA* OR MAOB*).

This search strategy identified publications that contain any one of these words and their derivatives in their title, abstract or keywords. No additional filters like publication year, document type (e.g., research article, review, editorial, and others), or publication language, were used.

### Data Extraction

The identified publications were evaluated for the following data: (1) publication year; (2) journal title; (3) total citation count; (4) authorship; (5) WoS category; and (6) manuscript type. The publication and citation data of authors, institutions, countries/regions, and journals were evaluated with the “Analyze” function of WoS. Then, we extracted the “full records and cited references” of these publications using the VOSviewer software (v.1.6.10, 2019). VOSviewer is a computer program that analyses the words within the titles and abstracts of the publications and produces a bubble map that illustrates their word frequency together with citation data (Van Eck and Waltman, 2010). Each bubble represents a keyword or a phrase. The bubble size indicates the keyword’s frequency (“n” represents multiple appearances in a publication count as one). The bubble color indicates the citation per publication (CPP) count for articles containing that keyword. Inter-bubble distance indicates frequency of co-occurrence or respective terms in publications. The term map visualizes terms that appeared in at least 199 (1.0%) of the included manuscripts. Another bubble map was similarly generated for author keywords that appeared in at least 20 (0.1%) of the publications.

### Visualization of Crystal Structures of MAOs

Visualization of the 3D crystal structures of the human MAO-A and human MAO-B enzyme was performed with the SeeSAR software (v.9.0, 2019 form BioSolveIT). SeeSAR enables quick and interactive assessments of the free energy of binding and torsions (Bietz et al., 2014). The crystal structure of the human MAO-A enzyme in complex with clorgyline (PDB code: 2BXS; De Colibus et al., 2005) and of the human MAO-B enzyme in complex with safinamide (PDB code: 2V5Z; Binda et al., 2007) were obtained from the Protein Databank (PDB) and used for visualization of the binding modes for the respective ligands (inhibitors). The HYDE scoring function as embedded in SeeSAR considers the free energy by computing the difference between the unbound and bond states. H-bonds (approximate enthalpy) and dehydration (“desolvation”, approximate entropy) effects of all non-hydrogen/heavy atoms (HA), contributing to the *overall* Gibbs free energy (ΔG) are computed with respect to their accessibility to water before and after binding (Betz et al., 2016). After HYDE computations that run for very few seconds, SeeSAR visualizes the (HYDE-) estimated free energy of binding (ΔG); spherical “coronas” ranging from dark red (unfavorable) to dark green (favorable for affinity) visualize the contribution of an atom and its environment to the overall free energy of binding; corona sizes correlate with the amount of contribution (Schneider et al., 2012).

The extracted data was also imported into CRExplorer (v.1.9, 2018), a computer program that performs cited reference analysis (Thor et al., 2016), and outputs the results as a “reference publication year spectroscopy” (RPYS) that shows a waveform with high peaks in years when the seminal references were published (Slotkin, 1999; Marx et al., 2014; Yeung and Wong, 2019; Yeung et al., 2019c). For instance, the articles published in years 1926, 1927, 1928, 1929, and 1930 were cited by the publications within the dataset 45, 52, 146, 56, and 55 times, respectively. The 5-year median value was 55. Therefore, on the waveform there was a positive peak in 1928 with a magnitude of 91 (because the citation count for 1928 was 146, which positively deviated from its 5-year median by 91). We only considered references with >10% contributions to positive peaks with magnitude >50. We recorded their citations received from publications within the dataset and total citations as recorded by WoS.

## Results and Discussion

**Figure 4:**
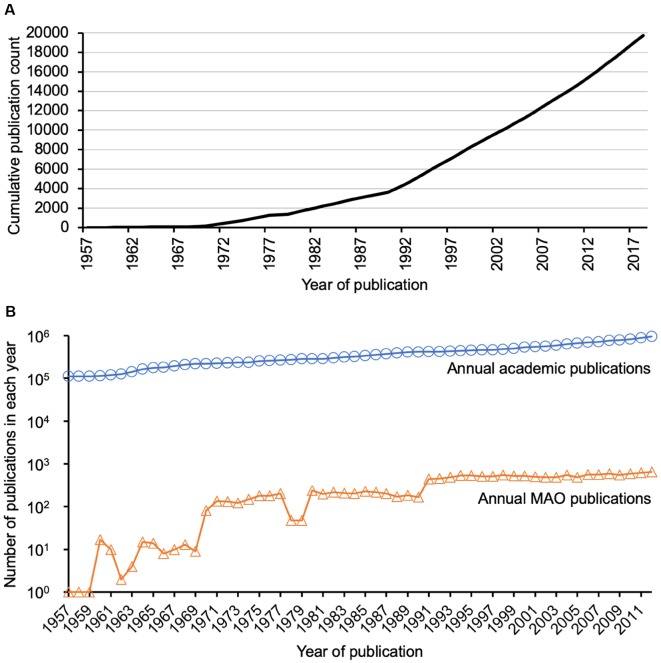
Publication trend. **(A)** Publication trend of monoamine oxidase (MAO) publications. There has been a continuous research interest (apparent as a linear growth of publication counts) since the 1990s. **(B)** Detailed comparison of annual total academic publications and annual MAO publications. The former was extracted from PubMed database using MEDSUM (http://webtools.mf.uni-lj.si/public/medsum.html), because Web of Science does not allow such a search query.

In general, the literature search resulted in 19,854 publications, which were released in the period of time between 1928 and March 2019. Figure 4 illustrates the continuous linear growth in MAO publications since the 1990’s. The limited number of publications before the 1990’s could be partly because of a lack of recording by WoS. The majority of the publications were original articles (*n* = 15,148, 76.3%) and reviews (*n* = 2,039, 10.3%). The remaining number of publications includes mainly meeting abstracts (*n* = 1,424), but also proceedings (*n* = 865), and brief articles (*n* = 378). The publications were mainly written in English (*n* = 19,099, 96.2%). The top five WoS categories of the analyzed MAO publications were Pharmacology/Pharmacy (*n* = 4,664, 23.5%), Neurosciences (*n* = 4,416, 22.2%), Psychiatry (*n* = 2,906, 14.6%), Biochemistry/Molecular Biology (*n* = 2,691, 13.6%), and Clinical Neurology (*n* = 1,754, 8.8%). This distribution was different for other topics such as anti-vascular endothelial growth factor (Yeung et al., 2019a) or antioxidant literature (Yeung et al., 2019b), in both of which Pharmacology/Pharmacy ranked third (7.3% and 11.8%, respectively); though these analyses also showed that original articles were the major publication type followed by review articles. The publications were contributed by over 46,000 authors from more than 7,200 institutions in 125 countries/regions and published in over 3,200 journals. All these data suggested the broad scientific community and importance of MAO research filed for neuroscience worldwide.

The top 10 most prolific authors have published at least 88 articles each, with CPP counts between 30.5 and 65.3 (Table 1). The top 10 institutions are scattered in the United States, UK, France, Sweden, Canada, Israel, and Russia, with CPP counts ranking from 9.2 to 54.3 (Table 2). The top 10 countries/regions with the most intensive research on the field of MAOs are the United States, followed by European and Asian countries (Table 3). In terms of CPP, China and India were lagging behind the other eight countries. The huge publication shares of these countries are similar to the pattern observed in the scientific literature of neuroscience in general (Yeung et al., 2017; Yeung, 2018).

**Table 1:** The top 10 contributing authors.

Author	Number of publications (% of total)	Citations per publication
Moussa B.H. Youdim	247 (1.2%)	55.6
Lars Oreland	232 (1.2%)	34.8
Jean C. Shih	143 (0.7%)	53.4
Keith F. Tipton	117 (0.6%)	47.4
Dennis L. Murphy	107 (0.5%)	65.3
Neal Castagnoli Jr.	102 (0.5%)	36.5
Kevin Chen	102 (0.5%)	57.2
Merton Sandler	95 (0.5%)	33.2
Vivette Glover	91 (0.5%)	30.5
Peter Riederer	88 (0.4%)	46.1

**Table 2:** The top 10 contributing institutions.

Institution	Number of publications (% of total)	Citations per publication
National Institutes of Health (NIH USA)	528 (2.7%)	48.5
University of California	463 (2.3%)	48.6
University of London	349 (1.8%)	50.5
French National Institute of Health and Medical Research (INSERM)	321 (1.6%)	38.2
Harvard University	307 (1.5%)	54.3
Uppsala University	278 (1.4%)	27.5
University of Toronto	273 (1.4%)	34.3
French National Center for Scientific Research (CNRS)	270 (1.4%)	32.0
Technion–Israel Institute of Technology	266 (1.3%)	51.2
Russian Academy of Sciences	248 (1.2%)	9.2

**Table 3:** The top 10 contributing countries.

County	Number of publications (% of total)	Citations per publication
USA	6,050 (30.5%)	37.4
UK	1,519 (7.7%)	35.3
China	1,374 (6.9%)	14.6
Japan	1,359 (6.8%)	20.9
Germany	1,219 (6.1%)	29.0
Italy	1,091 (5.5%)	31.5
Canada	1,058 (5.3%)	31.3
France	969 (4.9%)	28.3
Spain	794 (4.0%)	26.3
India	732 (3.7%)	14.9

Most of the top 10 journals were specialized in pharmacology and neuroscience. Among them, *Journal of Neurochemistry* had the highest publication and CPP counts (Table 4).

**Table 4:** The top 10 contributing journals.

Journal	Number of publications (% of total)	Citations per publication
Journal of Neurochemistry	340 (1.7%)	50.8
Biochemical Pharmacology	272 (1.4%)	29.8
Journal of Neural Transmission	227 (1.1%)	22.6
Brain Research	215 (1.1%)	29.4
Biological Psychiatry	185 (0.9%)	38.3
European Journal of Pharmacology	183 (0.9%)	27.9
Life Sciences	176 (0.9%)	29.1
Neuroscience Letters	168 (0.8%)	19.7
British Journal of Pharmacology	167 (0.8%)	33.9
Psychopharmacology	153 (0.8%)	37.0

**Figure 5:**
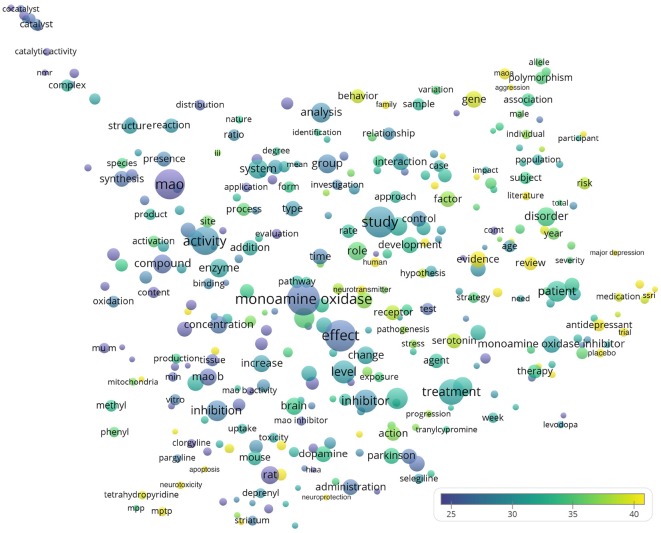
Bubble map of words from titles and abstracts of the 19,854 monoamine oxidase publications. Words from titles and abstracts were analyzed and visualized using the VOSviewer software (v.1.6.10, 2019). The map shows around 335 terms that appeared in at least 1.0% (199) of publications. Each bubble represents a term or phrase. Larger bubbles represent words (or terms) that appeared more frequently. More yellowish bubbles represent words that appeared in publications with more citations. Bubbles in closer proximity represent words (or terms) that co-appeared more frequently.

Figure 5 shows the words appearing in the title and abstracts of all analyzed 19,854 publications. Among the largest bubbles, several keywords were represented such as treatment (*n* = 4,189; CPP = 30.9), disease (*n* = 2,957; CPP = 31.9), inhibitor (*n* = 3,370; CPP = 28.6), and monoamine oxidase inhibitor (*n* = 1,915; CPP = 30.3). Meanwhile, examples of words with highest CPPs included reactive oxygen species (ROS; *n* = 239; CPP = 50.4), major depression (*n* = 233; CPP = 48.9), SSRI (selective serotonin reuptake inhibitor, *n* = 232; CPP = 46.8), aggression (*n* = 282; CPP = 45.6), substantia nigra (*n* = 268; CPP = 44.9), apoptosis (*n* = 252; CPP = 41.4), neuroprotection (*n* = 233; CPP = 40.2), and neurotoxicity (*n* = 406; CPP = 40.0).

**Figure 6:**
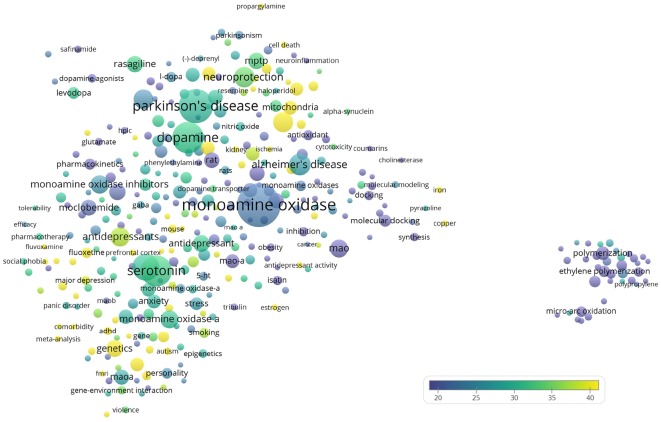
Bubble map of author keywords of the 19,854 monoamine oxidase publications. Author keywords were analyzed and visualized using the VOSviewer software (v.1.6.10, 2019). The map shows 348 keywords that appeared in at least 0.1% (20) of publications. Each bubble represents an author keyword. Larger bubbles represent keywords that appeared more frequently. More yellowish bubbles represent keywords that appeared in publications with more citations. Bubbles in closer proximity represent keywords that co-appeared more frequently.

**Figure 7:**
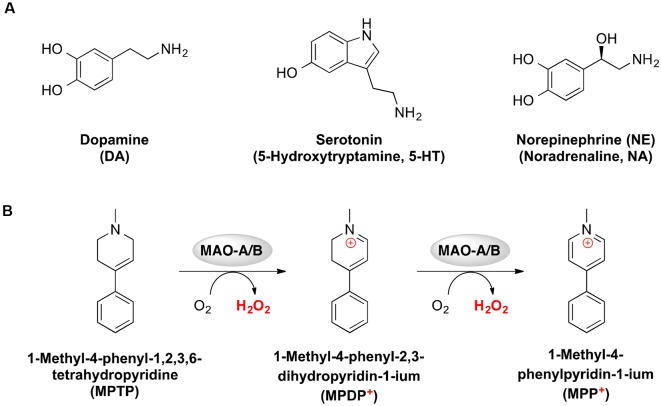
Chemical structures of neurotransmitters **(A)** and metabolism of 1-methyl-4-phenyl-1,2,3,6-tetrahydropyridine (MPTP) toward the neurotoxin MPP^+^ **(B)**, which were identified as recurring themes for monoamine oxidase studies.

The author keywords of the publications are visualized in Figure 6. The most frequently mentioned medical conditions and associated with such conditions chemicals/pharmaceuticals are listed in Tables s 5, 6, respectively. PD, depression and AD were most frequently mentioned, which are consistent to previous analyses showing that PD and AD are among the most intensively investigated medical conditions in neuropharmacology (Yeung et al., 2018). The frequently mentioned chemicals/pharmaceuticals involved common neurotransmitters, such as DA, serotonin (5-hydroxytryptamine, 5-HT), and (NE, also known as noradrenaline, NA), MAOIs like selegiline, rasagiline, moclobemide, and clorgyline, as well as 1-methyl-4-phenyl-1,2,3,6-tetrahydropyridine (MPTP), which is a prodrug of 1-methy4-phenylpyridinium (MPP^+^), a mitochondrial neurotoxin leading to destruction of glial cells in *substantia nigra* and, therefore, associated with PD (Table 6, Figures 3, 7; Kopin, 1987; Edmondson et al., 2009; Tripathi et al., 2018). Furthermore, it is also suggested that *substantia nigra* is rich in DA, which may undergo enzymatic oxidation *via* the MAO-B enzyme to form ROS, which plays a key role in the development of PD (Fahn and Cohen, 1992; Jenner and Olanow, 1996). It is believed that the high activity of MAO-B will increase the peroxidative stress that similarly contributes to the formation of AD (Benzi and Moretti, 1995). Therefore, MAOIs and in particular reversible MAO-B inhibitors, have been extensively evaluated for their neuroprotective effects as single therapeutics or in combination with other medications for the treatment of AD and PD, while selective reversible MAO-A inhibitors were successfully developed as antidepressants (Sano et al., 1997; Riederer et al., 2004a; Youdim and Bakhle, 2006). Many investigations have been conducted along these research directions, and thus accumulating publication counts. In recent years, dual inhibition of MAO-A/B is considered as privileged strategy in addition to other biological effects for the development of so called multi-target-directed ligands (MTDLs) for the treatment of neurodegenerative diseases, in particular, AD (Kumar et al., 2016; Tzvetkov and Atanasov, 2018). In addition, some research articles deal with the investigation of the key role of MAOs as potential drivers in non-neurological disorders, for example, mitochondrial dysfunctions associated with cardiac aging (Sheydina et al., 2011; Maggiorani et al., 2017).

**Table 5:** Medical and mental conditions mentioned in the author keywords of 0.5% (*n* = 100) of the monoamine oxidase publications.

Medical condition	Number of publications (% of total)	Citations per publication
Parkinson’s disease	789 (4.0%)	29.9
Depression	483 (2.4%)	30.2
Alzheimer’s disease	316 (1.6%)	26.6
Anxiety	134 (0.7%)	29.0
Schizophrenia	125 (0.6%)	30.1
Aggression	120 (0.6%)	43.0

**Table 6:** Chemicals and pharmaceuticals mentioned in the author keywords of 0.5% (*n* = 100) of the monoamine oxidase publications.

Chemical/pharmaceutical	Number of publications (% of total)	Citations per publication
Dopamine	665	31.7
Serotonin	649	32.1
Selegiline	257	24.4
MPTP (1-methyl-4-phenyl-1,2,3,6-tetrahydropyridine)	180	32.8
Rasagiline	178	32.1
Moclobemide	173	21.2
Norepinephrine	113	26.7
Clorgyline	110	22.9

**Figure 8:**
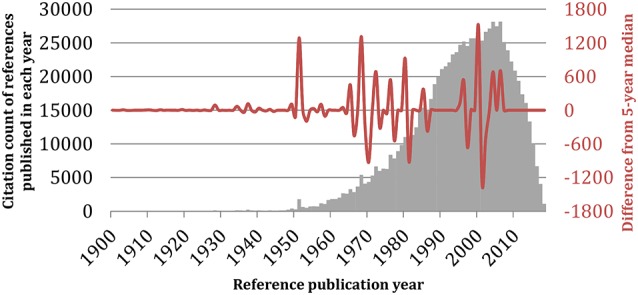
Reference publication year spectroscopy (RPYS) method. The reference lists of the 19,854 monoamine oxidase publications were analyzed using the CRExplorer software (v.1.9, 2018). References were sorted by publication year (horizontal axis), and the citation counts received by all references published in the same year were added up and visualized in bars (gray vertical axis). The waveform showed the fluctuations in the deviation of the annual citation count from its 5-year median (red vertical axis).

Furthermore, we performed an analysis of the citation count of references per year for the whole investigated period of time 1928–2019. For this purpose, we applied the RPYS method as embedded in CRExplorer (Thor et al., 2016). Figure 8 shows the RPYS plot for the cited reference analysis for each year and for each publication. Following the RPYS method, 16 positive peaks with magnitude >50 for 17 separate years were found, as follows: 1928, 1934, 1937, 1949, 1951, 1957, 1965, 1968, 1972, 1976, 1980, 1985, 1995–1996, 2000, 2004, and 2006. From the figure, it seemed that the gray bars formed a bell that centered on the 2000–2010 years, meaning that many cited references were published during these years. Potential explanations could be that in those years the recombinant forms of MAO enzymes were available and the crystal structure solved, thus these important references were more cited. Meanwhile, there was a strong positive peak in 2000. Some references were cited >100 times by the analyzed MAO publications, though they did have >10% contributions to the peak. Two such exemplars were reporting inhibition of MAO by coumarin derivatives (Gnerre et al., 2000), and a potential association between MAOA gene polymorphism and variability in aggressiveness and impulsivity (Manuck et al., 2000). Nine seminal references fulfilled our predefined criteria of having >10% contributions to a positive peak with magnitude >50. All of them were published in 1972 or before (Table 7). The seminal paper by Mary Bernheim (Hare, 1928) was identified in the first positive peak. Another notable seminal reference was by Lowry et al. (1951), which described the technique of a biochemical assay to measure the total level of protein in a test solution. It should be noted that by analyzing the cited references, we were able to identify not only the historical root of MAO but also articles reporting methods that became standard techniques in protein research field and thus highly cited by the MAO field. If only seminal papers regarding the MAO proteins were focused on, then these references were identified in four representative years: 1928, 1957, 1968, and 1972.

**Table 7:** Seminal references of monoamine oxidase publications identified by the RPYS method.

Year	References	% Contribution to the peak	Citations by MAO publications	All-time total citations
1928	Hare (1928)	65.1	95	318*
1934	Lineweaver and Burk (1934)	50.3	77	12,422*
1937a	Blaschko et al. (1937a)	20.1	45	473*
1937b	Blaschko et al. (1937b)	10.7	24	197*
1949	Gornall et al. (1949)	16.2	62	16,989
1951	Lowry et al. (1951)	80.0	1,437	3,37,283
1957	Glenner et al. (1957)	12.0	140	685
1968	Johnston (1968)	22.5	1,218	1,596
1972	Knoll (1972)	10.3	688	1,014*

*Citation count recorded from Google Scholar, as the publication is not indexed by Web of Science.

The current analysis has some limitations. First, the search strategy might limit the body of literature to be analyzed. Second, some MAO articles might not be indexed by the WoS database, especially the older ones. Alternative databases could be considered, such as Scopus, to identify additional publications, but data from multiple databases cannot be merged due to their differences in indexing and counting, and thus cannot be integrated in our analysis. Readers should also be aware of the general increase of scientific production along the 20th century (Figure 4B), which was growing in a higher level compared to MAO publications in recent years (until 70s the trend was the opposite and the number of MAO articles was increasing more rapidly than the total number of academic articles). Nevertheless, the graph shows that the interest in these proteins is still very high. In general, the number of scientific articles, citations, journals, and institutions has been also progressively increasing. It would be interesting to normalize the results (publication and citation data of the MAO research field) with respect to these changing parameters. However, such normalization would be very complicated and hence not applicable for the current study. To the best of the authors’ knowledge, a similar bibliometric analysis approach has not been done before so that in the future it can be applied to other targets (e.g., specific proteins) to allow a better comparison.

In conclusion, the current MAO literature analysis highlights the popular research themes in the scientific literature related to MAOs and historical roots of MAO research as a quick guide for fellow researchers. Through decades of research, the literature has accumulated many publications investigating the therapeutic effects of at least two generations of MAOIs on various neurological conditions, such as AD, PD, and depression. The analyzed data showed that the United States is the major contributor, together with some European and Asian countries. Many of the articles were published in pharmacology and neuroscience journals. Publications involving the neurotransmitters DA, serotonin, and NE, as well as the MAO-A inhibitors moclobemide and clorgyline, and the irreversible MAO-B inhibitors selegiline and rasagiline had over 20 citations per publication. We envision that the number of publications related to MAOs research will continue to grow steadily, with more new drugs being tested for better management of neurological conditions, in particular, with the development of multi-target acting drugs against neurodegenerative diseases. Moreover, the analysis of the scientific literature suggested that in addition to the pivotal role of MAOs as biological targets in neuroscience, the research field will also be directed toward investigations of MAOIs as potential therapeutics for other pathophysiological processes associated with aging, such as increased sensitivity to apoptosis, increased production of mitochondrial ROS, and others.

## Data Availability

The datasets generated for this study are available on request to the corresponding author.

## Author Contributions

All authors conceived the work. AY and NT acquired data and drafted the work. AY and AA analyzed data. AA, MG and NT critically revised the work. All authors have approved the final content of the manuscript.

## Conflict of Interest Statement

The authors declare that the research was conducted in the absence of any commercial or financial relationships that could be construed as a potential conflict of interest.
